# The multifaceted effects of fluoxetine treatment on cognitive functions

**DOI:** 10.3389/fphar.2024.1412420

**Published:** 2024-07-16

**Authors:** Estíbaliz Ampuero, Alejandro Luarte, Francisca Sofia Flores, Antonia Ignacia Soto, Catalina Pino, Viviana Silva, Macarena Erlandsen, Teresita Concha, Ursula Wyneken

**Affiliations:** ^1^ Laboratorio Neurofarmacología del Comportamiento, Facultad de Química y Biología, Universidad de Santiago, Santiago, Chile; ^2^ Laboratorio Neurociencias, Facultad de Medicina, Universidad de los Andes, Santiago, Chile; ^3^ IMPACT, Center of Interventional Medicine for Precision and Advanced Cellular Therapy, Santiago, Chile

**Keywords:** antidepressants, SSRIs, side-effects, memory, cognition

## Abstract

Fluoxetine, the prototypical selective serotonin reuptake inhibitor (SSRI), is widely used to treat major depressive disorder (MDD) and a variety of other central nervous system conditions, primarily due to its established clinical safety profile. Although its efficacy in treating depression is well-recognized, the impact of fluoxetine on cognitive functions remains inconsistent and elusive. In this review, we first examine the well-substantiated biological mechanisms underlying fluoxetine’s antidepressant effects, which include serotonin reuptake inhibition and activation of TrkB receptors—key to brain-derived neurotrophic factor (BDNF) signaling. Subsequently, we delve into the cognitive side effects observed in both preclinical and clinical studies, affecting domains such as memory, attention, and executive functions. While certain studies indicate cognitive improvements in patients with underlying disorders, there is also evidence of negative effects, influenced by variables like gender, duration of treatment, age, disease pathology, and the specifics of cognitive testing. Significantly, the negative cognitive outcomes reported in preclinical research often involve healthy, non-diseased animals. This review underscores the necessity for heightened caution in fluoxetine prescription and further investigation into its potentially detrimental cognitive effects, even when used prophylactically.

## Introduction

Embarking on a quest to mitigate the burden of major depressive disorder (MDD), a pervasive mental health challenge, scientists heralded a new era in the 1950s with the discovery of imipramine, the archetype of tricyclic antidepressants. This breakthrough paved the way for a novel class of therapeutics. In this vein, fluoxetine emerged from an extensive search to find antidepressants that sidestepped the high affinity for monoaminergic neurotransmitter receptors, a common source of the undesirable side effects linked with their tricyclic counterparts (Beaumont, 1988). From a pool of 55 phenoxyphenylpropylamine derivatives assessed for their ability to inhibit monoamine uptake, one, in particular, stood out by 1972: fluoxetine sodium oxalate distinguished itself as a potent and selective serotonin reuptake inhibitor (SSRI), charting a new course in antidepressant treatment (Wong et al., 1995; Wong and Licinio, 2001; Wong et al., 2005; Lopez-Munoz and Alamo, 2009).

*In vivo* studies have delineated the role of fluoxetine in modulating synaptic serotonin (5-HT) levels, revealing a modest yet persistent surge in extracellular 5-HT concentrations (Guan and McBride, 1988; Rutter et al., 1994; Invernizzi et al., 1996; Tao et al., 2000; Koch et al., 2002; Wong et al., 2005). Its bioactive metabolite, norfluoxetine, also elevates 5-HT levels post-treatment (Qu et al., 2009). Despite diminished interactions with monoamine receptors, fluoxetine still has interactions with 5-HT2A and 5-HT2C receptors, displaying a spectrum of signaling from agonist at 5-HT1A/2A receptors to antagonist at 5HT2C and 5-HT2A receptors (Ni and Miledi, 1997; Bampalis et al., 2010; Liu L. et al., 2018). Such findings highlight the complex role of serotonin receptor activity in shaping fluoxetine’s pharmacological action. There is a strong imperative for more detailed research into fluoxetine’s genetic and cellular mechanisms and its influence on the dynamics of neuronal networks. A thorough understanding of these processes is crucial for maximizing fluoxetine’s therapeutic benefits and reducing its adverse effects, representing a pivotal challenge in the advancement of antidepressant therapy.

### Mechanisms of fluoxetine’s therapeutic action

Fluoxetine is known to rapidly increase synaptic 5-HT levels, but its therapeutic effect is mediated by plastic changes within neuronal networks that require weeks or even months to establish (Saarelainen et al., 2003; Wyneken et al., 2006). The neurotrophin Brain-Derived Neurotrophic Factor (BDNF) and its receptor, tropomyosin kinase B (TrkB), are believed to play a crucial role in inducing these plastic adaptations (Nibuya et al., 1995; Nibuya et al., 1996; Saarelainen et al., 2003; Wyneken et al., 2006; Martinowich et al., 2007; Rantamaki et al., 2007; Rantamaki et al., 2011; Mikics et al., 2018). Indeed, BDNF is known to be involved in processes critical to neuronal health, including development, survival, neurogenesis, and synaptic plasticity (Reichardt, 2006; Leal et al., 2015). There is substantial evidence indicating that following several weeks of fluoxetine treatment, as well as other antidepressants, there are significant changes in the BDNF signaling pathway and downstream molecules such as Erk1/2 and, more recently, NF-κB (Castren, 2004; Molteni et al., 2006; Castren et al., 2007; Castren and Rantamaki, 2010; Huang et al., 2013; Yang et al., 2014; Cui et al., 2016; Mikics et al., 2018; Song et al., 2019). An intriguing finding by Casarotto et al. indicates that the antidepressant effect of fluoxetine is reliant on its direct binding to the TrkB receptor, which facilitates synaptic localization of TRKB and its activation by BDNF (Casarotto et al., 2021). Thus, different experimental approaches suggest that enhancements in BDNF/TrkB signaling are fundamental to the activity-dependent synaptic plasticity that leads to mood improvements due to fluoxetine treatment (Stewart and Reid, 2000; Maya Vetencourt et al., 2008; Wang et al., 2008; Guirado et al., 2014; Ruiz-Perera et al., 2015; Mikics et al., 2018). Furthermore, transactivation of the TrkB receptor by external factors such as oxytocin or exercise, possibly acting through this same signaling system, is suggested to augment the therapeutic action of fluoxetine and other antidepressants (Sleiman et al., 2016; Mitre et al., 2022), which may constitute an innovative and complementary approach for depression treatment (Madjid et al., 2023).

BDNF also plays a critical role in adult neurogenesis, particularly within specialized regions of the brain known as adult neurogenic niches. One such niche is the subgranular zone of the hippocampus, where BDNF facilitates the terminal differentiation of new neurons (Chan et al., 2008). The significance of adult neurogenesis in the mechanism of antidepressants has been well established, with numerous studies documenting this relationship (Waterhouse et al., 2012; Quesseveur et al., 2013; Bjorkholm and Monteggia, 2016; Tunc-Ozcan et al., 2019; Malberg et al., 2021). The positive effect on neurogenesis of several antidepressants converges on bone morphogenetic protein (BMP), a central regulator of neural stem cell proliferation, and BDNF/TrkB is proposed to be one of the implied signaling systems (Tunc-Ozcan et al., 2021). Fluoxetine has been shown to stimulate neurogenesis, which is not only associated with its therapeutic effects but also counteracts the neurogenesis decline caused by chronic stress (Santarelli et al., 2003; Tanti et al., 2012). This restorative effect on neurogenesis is linked to increases in BDNF and involves a 5-HT4 receptor-dependent de-maturation of granule cell populations (Imoto et al., 2015). Interestingly, fluoxetine also promotes the neurogenesis of interneurons in the cerebral cortex, a process that is coupled with an inhibitory effect on ischemia-induced apoptosis (Ohira et al., 2013). Currently, the relationship between fluoxetine’s neurogenesis-enhancing effects and its ability to selectively induce structural and synaptic changes that alleviate depressive symptoms is not fully understood and warrants further investigation.

Fluoxetine treatment elicits morphological and synaptic changes in neuronal networks, whose beneficial effects may depend on factors like age, brain region, and physiological status. As such, fluoxetine treatment induces a reduction in dendritic arbor length and complexity in the cortical regions of adult animals (Ampuero et al., 2019), while these parameters are enhanced in the dentate gyrus of young animals, suggesting age and brain region-specific therapeutic window for both parameters (Amellem et al., 2017). At the synaptic level, fluoxetine treatment increases dendritic spine density in the hippocampus and medial prefrontal cortex (Ampuero et al., 2010; Rubio et al., 2013; McAllister et al., 2015; Pedraza et al., 2019; Song et al., 2019). In naïve animals, repetitive fluoxetine treatment is characterized by the development of robust and larger spines, which are enriched with AMPA and GluN2A-type NMDA receptor subunits, known to be prevalent in mature dendritic spines, and associated with enhanced synaptic responses (Popoli et al., 2002; Ampuero et al., 2010; Barbon et al., 2011; Rubio et al., 2013). This type of synaptic maturation, however, is linked to altered or diminished long-term potentiation (LTP) and long-term depression (LTD), both of which are extensively validated models of synaptic plasticity (Stewart and Reid, 2000; Rubio et al., 2013). These changes imply that, under physiological or healthy conditions, repetitive fluoxetine treatment leads to long-term stabilization and decreased plasticity of neuronal networks that might be relevant for learning and cognition. However, the induction of such plastic adaptations may be beneficial in restoring the synaptic balance disrupted during pathological states, including prolonged stress and mood disorders (Kallarackal et al., 2013; Mikics et al., 2018). Furthermore, beneficial effects of fluoxetine’s plasticity-promoting actions may extend to other pathological conditions such as neurodegenerative diseases or injury. In Alzheimer’s disease mice models, for example, fluoxetine treatment has been shown to increase dendritic spine numbers and elevate levels of synaptic proteins such as postsynaptic density 95 (PSD-95) and synapsin-1 in the hippocampus, indicative of a beneficial increase in synapse formation and stability (Ma et al., 2020). Similarly, following medial frontal cortex injury, fluoxetine led to an increase in dendritic spine density and basal dendritic arborization in layer II/III pyramidal neurons (McAllister et al., 2015). The exact role of BDNF or its downstream pathways in mediating these fluoxetine-enhanced plastic changes remains to be fully elucidated.

The long-term implications of fluoxetine’s capacity to promote neural plasticity, beyond its antidepressant effects, remain unclear and under documented. Establishing clear causal links between prolonged fluoxetine use and its potential side effects in relevant domains such as cognitive function is essential. Next, we will describe evidence regarding the detrimental and positive effects of fluoxetine in preclinical models of depression and in humans.

### Cognitive side effects of fluoxetine

Fluoxetine is primarily used to treat major depressive disorder (MDD), a disease that is often accompanied by impaired cognitive functions, a complex concept globally including memory, executive functions, attention, and psychomotor processing speed (Wagner et al., 2012; Hammar et al., 2022). Moreover, cognitive deficits may persist in treated patients that recover from depressive symptoms, and thus, the contribution of antidepressant treatment to persistence, aggravation or improvement on cognition is difficult to grasp. Regarding cognitive functions, this the first time that preclinical findings and clinical findings are summarized side-by-side in adults receiving fluoxetine. Recent reviews have addressed the most commonly studied side effects (but not cognition) in children and adolescents (an age group that was excluded in the present review) (Locher et al., 2017). We also excluded pre-natal and perinatal fluoxetine treatments. One of the reviews on adults focuses on suicide ideation, which cannot be studied in pre-clinical models (Edinoff et al., 2021). In one recent review, the levels of BDNF in preclinical and clinical studies are analyzed side-by-side. Interestingly, conflicting results were found in animal studies while in humans, the anti-depressant effect of drugs (including fluoxetine) seems not to depend on BDNF (Talaee et al., 2024). We now summarize the available literature in the field focusing first on animal models and second, on studies with patients, and separating detrimental effects (Table 1) from positive effects (Table 2). Excellent reviews have examined preclinical studies, which, to some extent, address the time and ethical constraints inherent in human studies (Monleon et al., 2008; Olivier et al., 2011). The explanation of the cognitive test used are contained in Box 1, and the clinical tests that have a preclinical counterpart are marked * (to see Supplementary material).

**TABLE 1:** Detrimental effects of fluoxetine on cognitive function as evaluated in preclinical and clinical studies.

Clinical	Model	Methodology	Age (months, at beginning of experiment)	Host	Treatment time (weeks); dose	References
	naïve	one-trial appetitive learning task	2	rats, male	acute 1, 3, 10 mg/kg	Lalonde and Vikis-Freibergs (1985)
	naïve	OLM; NOR; MWM	2	rats, male	4 0.7 mg/kg	Ampuero et al. (2013)
	naïve	NOR	3	mice, male	acute 10 mg/kg	Castane et al. (2015)
	Dentate gyrus lesion	MWM	2	rats, male	6 5 mg/kg	Keith et al. (2007)
	naïve (**male affected**)	IAT	4	mice, male and female	3 20 mg/kg	Monleon et al. (2002)
	naïve prenatal stress	NOR; MWM	3	rats, male	9 + 2 0.7 mg/kg	Moraga-Amaro et al. (2021)
	naïve	NOR; appetitive conditioning; MWM no effect	2	rats, male	2 5 mg/kg	Valluzzi and Chan (2007)
	naïve	IAT, open arm escape	2	mice, male	acute, and 2 40 mg/kg	Gomes et al. (2009)
	naïve **(male affected)**	IAT	1.5	mice, male and female	acute 15 mg/kg	Parra et al. (2010)
	naïve	MWM	3	rats, male	acute 8 and 16 mg/kg	Majlessi and Naghdi (2002)
	naïve (review)	IAT, AAT, MWM	2 to 4	mice and rats	pre-training acute and 2 0.25–30 mg/kg	Monleon et al. (2008)
	naïve	AAT	2	rats, male	acute 10 mg/kg	Nelson et al. (1997)
	naïve	NOR	PND 21	rats, male and female	6 5 mg/kg	Sadegzadeh et al. (2020)
	naïve	CPT	12	monkeys, male	96 2 mg/kg	Golub et al. (2018)
	naïve	impulsivity test (Wisconsin)	12	monkeys, male	48	He et al. (2014)
	naïve	reward learning	3	mice, female	8 10 mg/kg	Puscian et al. (2021)
	addiction (methylphenidate)	cocaine self-administration	2.5	rat, male	1 5 mg/kg	Lamoureux et al. (2023)
	naïve	cued fear conditioning	2	mice, male	acute and 3 18 mg/kg	Payet et al. (2023)

Clinical	Pathological condition	Methodology	Age (years)	Patients, number	Treatment time (weeks); dose	References
	Firstly diagnosed with depression	Quantitative clinical memory scale Ruff 2&7 Selective Attention Test	adult	102	12 20, 40 mg/kg	Luo et al. (2013)
	Depression	MMSE	87	case report	9 20 mg/kg	Joss et al. (2003)
	Depression or OCD	MMSE	20–60	50	8 dose not reported	Sayyah et al. (2016)
	Depression	SMQ; Word Stem Completion No effect: SSDT	20–61	14	80 20–80 mg/kg	Gorenstein et al. (2006)
	Depression	WNVS; CANTAB	12–18	48	12 20, 40, 60 mg/kg	Shehab et al. (2016)
	Healthy volunteers	Mackworth Clock Test	21–45	18	3 20 mg/kg	Ramaekers et al. (1995)

Box 1 contains information relevant to cognitive domains evaluated in the applied tests. In preclinical studies, the columns are: Methodology, including the tests that were used to assess cognitive function. The tests that were not affected are also specified with “no effect:”; Age (months at the beginning of experiment); Host or species; Treatment time in weeks and fluoxetine dose; Reference. In clinical studies, the columns are: Pathological condition; Methodology including the tests that were used to assess cognitive function. The tests that were not affected are also specified with “no effect:”; Age in years; Number of Patients; Weeks of treatment and dose; Reference.

**TABLE 2:** Positive effects of fluoxetine on cognitive function as evaluated in preclinical and clinical studies.

Preclinical	Model	Methodology	Age (months, at beginning of treatment)	Host	Treatment time (weeks); dose	References
	naïve	amphetamine self administration	2	rats, male	3 days 5 mg/kg	Yu et al. (1986)
	naïve	cocaine self administration	3	rats, male	5 days 5 mg/kg	Glatz et al. (2002)
	naïve	OLM; Barnes maze test	1.5	mice, male	4 days, 1 15 mg/kg	Yi et al. (2018)
	naïve	OLM; CFC extinction	4.5	mice, male and female	1, 2 15 mg/kg	Casarotto et al. (2021)
	naïve	Place learning and reversal	2	mice, female	2 10 mg/kg	Marwari and Dawe (2018)
	Stroke model	MWM	3	mice, male	3–4 18 mg/kg	Vahid-Ansari and Albert (2018)
	Stroke model	MWM	2	mice, male	4 10 mg/kg	Li et al. (2009)
	Ischemia model	Y- maze	2	mice, male	1 10 mg/kg	Lee et al. (2014)
	Temporal lobe epilepsy model	MWM	1.5	rats male	6 20 mg/kg	Barkas et al. (2012)
	Alzheimer´s disease model, Social Isolation model	MWM	8	rats	5 10 mg/kg	Abu-Elfotuh et al. (2022)
	Alzheimer´s disease model	MWM	17–18	mice, male	5 10 mg/kg	Ma et al. (2017)
	Alzheimer´s disease model	MWM	6	mice, sex not reported	2 10 mg/kg	Jin et al. (2017)
	Alzheimer´s disease model	MWM; Y-maze	2	mice, male and female	28 2.5, 5 mg/kg	Wang et al. (2014)
	Cancer chemotherapy model	OLM; CFC	8	rats male	2 10 mg/kg	ElBeltagy et al. (2010)
	Cancer chemotherapy model	NOR	8	rats male	6 10 mg/kg	Lyons et al. (2011)
	Down Syndrome model	NOR; Y-maze	4	mice, male and female	8 10, 20, 40 mg/kg	Begenisic et al. (2014)
	Chronic mild stress + Aβ1–42	Y-maze	2	rats male	2 10 mg/kg	Zabot et al. (2024)
	naïve	visual system-dependent attention and reward tasks	12 years	rhesus macaques, male	4 2.5 mg/kg	Gacoin and Ben Hamed (2023)
	Chronic unpredictable mild stress	Context-dependent fear extinction	5–10	mice, sex not reported	1 10 mg/kg	Joo et al. (2024)
	Chronic unpredictable mild stress + LPS	MWM	1.5	mice, male	5 10 mg/kg	Li et al. (2023)
	Chronic unpredictable mild stress + LPS	NOR; no effect: CFC	3–4	mice, male	4 20 mg/kg	Liu et al. (2023)
	*Trypanosoma cruzi* infection	NOR	5–7	mice, female	4 10 mg/kg	Vilar-Pereira et al. (2024)
	Social defeat stress + ethanol	NOR; Y-maze	1.5	mice, male	2 10 mg/kg	Ben-Azu et al. (2024)
	Neonatal hypoxia	NOR; cognitive flexibility; no effect: OLM	2.5–4	mice, male and female	4 10 mg/kg	Lee et al. (2024)
	Sepsis-associated encephalopathy LPS	Barnes maze; no effect: NOR	2.5–3	mice, male	2 10, 20 mg/kg	Zhang et al. (2024)

Clinical	Pathological condition	Methodology	Age (years)	Number of Patients	Weeks of treatment; dose	References
	Mild cognitive impairment	MMSE; No effect: WMS-III	55–75	58	8 10, 20 mg/kg	Mowla et al. (2007)
	Traumatic brain injury	Trail Making Test; WAIS-III; No effect: MMSE, USC-REMT	43–50	5	32 20–60 mg/kg	Horsfield et al. (2002)
	Depression	PGIMS	15–55	31	2 and 4 20 mg/kg	Jaykaran et al. (2009)
	Depression	CANTAB	12 to 18	24	6 and 12 20, 30, 40 mg/kg	Maalouf et al. (2018)
	Depression	BSRT; BIMT; CTT; CLAS; WPW	74.85 ± 6.67	119	52 20–60 mg/kg	Cassano et al. (2002)
	Depression	SLT; DSST	67.4 ± 5.9	105	12 20, 40 mg/kg	Doraiswamy et al. (2003)
	Alcoholics (mild to moderate)	Experimental drinking sessions	19–59	16	2 60 mg/kg	Naranjo et al. (1994)
	Vascular dementia	MMSE; TPC; No effect: VFT; DSST	45–80	50	12 25 mg/kg	Liu et al. (2014)
	Vascular dementia	MMSE; TPC; No effect: VFT; DSST	45–80	47	12 20 mg/kg	Zhang et al. (2021)

AAT: Active Avoidance Test.

BIMT: Blessed Information and Memory Test.

BSRT: Buschke Selective Reminding Test.

CANTAB: Cambridge Neuropsychological Test Automated Battery.

CFC: Contextual Fear Conditioning.

CLAS: Clifton Assessment Schedule.

CTT: Cancellation Task Test.

CPT: Continuous Performance Test.

DSST: Digit Symbol Substitution Test, WAIS subtest.

IAT: Inhibitory Avoidance Test.

MMSE: Mini-Mental State Examination.

MWM: Morris Water Maze.

NOR: Novel Object Recognition.

OCD: Obsessive-Compulsive Disorder.

OLM: Object Location Memory.

PGIMS: The PGI Memory Scale.

SLT: Shopping List Task.

SMQ: Subjective Memory Questionnaire.

TPC: Ten-Point Clock Drawing score.

USC-REMT: University of Southern California Repeatable Episodic Memory Test.

VFT: Verbal Fluency Test.

WAIS-III: Wechsler Adult Intelligence Scale.

WMS-III: Wechsler Memory Scale III.

WNV: The Wechsler Non-verbal Scale of Ability.

WPW: Wechsler Paired Word Test.

Box 1 contains information relevant to cognitive domains evaluated in the applied tests. In preclinical studies, the columns are: Methodology, including the tests that were used to assess cognitive function. The tests that were not affected are also specified with “no effect:”; Age (months at the beginning of experiment); Host or species; Treatment time in weeks and fluoxetine dose; Reference. In clinical studies, the columns are: Pathological condition; Methodology including the tests that were used to assess cognitive function. The tests that were not affected are also specified with “no effect:”; Age in years; Number of Patients; Weeks of treatment and dose; Reference.

#### Detrimental cognitive effects of fluoxetine treatment in preclinical models (Preclinical)

In the search for learning and memory outcomes of fluoxetine treatment, both single-dose and sustained administration of this antidepressant (over 2 weeks) have been shown to impair long-term memory in naïve rats across a range of tests for measuring inhibitory avoidance, learning and impulsivity, while short-term memory is not impaired (refer to Table 1 for a detailed breakdown). However, despite rats treated with fluoxetine maintain their ability to learn the location of a submerged platform (Keith et al., 2007; Valluzzi and Chan, 2007; Satvat et al., 2012; Ampuero et al., 2013), their recall might be compromised after an extended period post-training (i.e., tested 17 days after training) (Ampuero et al., 2013), suggesting that fluoxetine elicits an specific impairment in remote memory. Also underscoring the complexity of fluoxetine treatment outcomes on cognition, hippocampal dependent tasks were not impaired during the training period while non-hippocampal tasks, such as novel object recognition were impaired in adult as well as adolescent animals (Valluzzi and Chan, 2007; Ampuero et al., 2013; Sadegzadeh et al., 2020), which may be associated to increased 5-HT2A receptor signaling in the prefrontal cortex (Lalonde and Vikis-Freibergs, 1985; Castane et al., 2015). Also, in the Barnes maze test, fluoxetine was not able to induce a recovery of the spatial memory deficits induced by traumatic brain injury (Wang et al., 2011). By the other hand, the prenatal administration of fluoxetine to pregnant females induces deficits in non-hippocampal memory in the adult rat (Moraga-Amaro et al., 2021). Regarding other relevant domains, chronic fluoxetine treatment induced sleep disruption, facilitated social interaction, but led to impairment of vigilance/sustained attention and enhanced impulsivity in juvenile rhesus monkeys (He et al., 2014; Golub et al., 2018) (To see Table 1).

#### Detrimental cognitive side effects of fluoxetine in patients (Clinical)

Considering the cognitive deficits observed in MDD patients, some studies address the outcomes of fluoxetine treatment on this issue (Table 1). In patients with a first diagnose of MDD, it was found that fluoxetine treatment reduces depressive symptoms, but it does not improve a specific memory scale, directional memory, free recall, associative learning, nor face recognition (Luo et al., 2013). Another cohort with MDD and obsessive-compulsive disorder patients, fluoxetine gradually and significantly decreased the score in the Mini-Mental State Examination (MMSE) test over 8 weeks of treatment (Luo et al., 2013; Sayyah et al., 2016; Shehab et al., 2016). In addition, deficits in the Subjective Memory Questionnaire were detected in MDD patients treated with fluoxetine compared to matched controls, both in remitted and non-remitted patients, while in objective memory tests no differences were found (Gorenstein et al., 2006). In aged patients a case report in an 85 years-old depressed patient showed a significant memory loss after the beginning of fluoxetine treatment, and this effect was reversed during drug withdrawal (Joss et al., 2003).This striking case suggest that potential the age-related sensitivity to fluoxetine’s cognitive effects should be spotlighted (To see Table 1).

Sustained attention and vigilance were also negatively affected in healthy adults treated with fluoxetine, underscoring the drug’s broader cognitive footprint beyond its therapeutic use (Ramaekers et al., 1995). Of note, a large number of studies indicate that the use of SSRIs in general and fluoxetine in particular elevate the likelihood of suicide attempts or fatalities, causing a continuous growth of apprehension regarding the use of fluoxetine especially among adolescents and young patients (Zhou et al., 2020; Lagerberg et al., 2022). The collective insights from these studies underscore an intricate tapestry of cognitive effects woven by fluoxetine treatment. It invites a deeper reflection on the balance between managing MDD and preserving cognitive integrity, especially in vulnerable populations.

#### Positive cognitive effects of fluoxetine in animal disease models (Preclinical)

Several preclinical studies have shown memory improvements after fluoxetine treatments. For instance, fluoxetine administration to naïve mice increases memory retention (Yi et al., 2018; Casarotto et al., 2021) and reverts memory deficits induced by neuropathological conditions such as in the social isolation model of depression (Abu-Elfotuh et al., 2022), Alzheimer disease (Wang et al., 2014; Jin et al., 2017; Ma et al., 2017; Abu-Elfotuh et al., 2022), ischemia (Chang et al., 2006; Lee et al., 2014), Down´s Syndrome (Begenisic et al., 2014), stroke (Li et al., 2009; Vahid-Ansari and Albert, 2018), epilepsy (Barkas et al., 2012), cancer chemotherapy (ElBeltagy et al., 2010; Lyons et al., 2011). These studies strongly suggest that in different brain pathological conditions, fluoxetine treatment positively affects the recovery of cognitive functioning. Conversely, healthy or control animals submitted to fluoxetine treatment are not necessarily benefited in their cognition, as fluoxetine-induced plastic changes might be shifting some neuronal networks out of a functional or homeostatic set point (To see Table 2).

However, the dose and timing of fluoxetine administration are important factors that may influence the impact of fluoxetine in the evaluation of cognitive functions under pathology. For instance, early life exposure of low doses of fluoxetine reduces the deficits in spatial memory induced by ischemic brain injury, while high doses of fluoxetine have no effects (Chang et al., 2006). Moreover, 28 days of fluoxetine treatment reduces the deficits on spatial memory induced after stroke by intraluminal middle cerebral artery occlusion in adult mice (Li et al., 2009). Depending on the animal model and experimental protocol, fluoxetine can exert opposing effects on cognitive functions: only short-term administrations (1 dose-up to 2 weeks) in naïve rats causes improvements in memory tests (Marwari and Dawe, 2018; Yi et al., 2018; Casarotto et al., 2021). Thus, while fluoxetine has shown cognitive benefits in various models of brain pathology, its effects in healthy systems and the optimal therapeutic window for its administration remain areas ripe for further inquiry. These findings not only expand our understanding of fluoxetine’s therapeutic scope but also caution against oversimplified applications, advocating for a tailored approach in its clinical use.

#### Positive cognitive effects of fluoxetine in patients (Clinical)

Chronic fluoxetine administration in patients with MDD has been associated with cognitive enhancements. Notably, adults on fluoxetine show improved recall memory and sharper attention and concentration, with benefits persisting even a month post-treatment (Jaykaran et al., 2009) (To see Table 2). Similarly, elderly MDD patients on long-term fluoxetine therapy exhibit cognitive improvements on memory assessments (Cassano et al., 2002; Doraiswamy et al., 2003). In adolescents, cognitive improvement in fluoxetine-treated depressed patients was shown (Maalouf et al., 2018). However, in a different controlled study performed in adolescents, 6 and 12 weeks of fluoxetine treatment improved executive functioning, sustained attention, inhibition, and impulsivity and executive functioning in healthy controls while in depressed patients, cognitive deficits were maintained (Shehab et al., 2016).

Beyond MDD, fluoxetine shows potential in ameliorating cognitive deficits related to other conditions. For example, individuals with traumatic brain injury (TBI) display improved working memory when treated with fluoxetine, as measured by the WAIS-III (Horsfield et al., 2002). Patients with mild cognitive impairment, often considered an early stage of Alzheimer’s disease, also benefit from fluoxetine, exhibiting better outcomes on the MMSE memory test than those on a placebo (Mowla et al., 2007). Moreover, those suffering from vascular dementia have shown cognitive improvements under fluoxetine treatment (Liu et al., 2014; Zhang et al., 2021). Collectively, these findings underscore fluoxetine’s significant role in reversing cognitive dysfunctions across various pathological conditions, bolstering memory, attention, planning, and concentration.

### Other reported side effects of fluoxetine

The weak interaction of fluoxetine with other neurotransmitter receptors explains its high tolerability profile. Its most common side effects include nausea and anorexia (Oliva et al., 2021). Beyond the gastrointestinal discomfort, a range of non-gastrointestinal side effects have been documented, such as headache, dizziness, variations in sleep patterns, anxiety, tremor, and sexual dysfunction, painting a comprehensive picture of the drug’s tolerability (Benfield et al., 1986; Ferguson, 2001; Carr and Ensom, 2002; Wernicke, 2004). Behavioral side effects, however, present a more elusive challenge, with detection proving more difficult and fewer studies available for reference. The translation of findings from animal models to human subjects adds an additional layer of complexity to the analysis of cognitive side effects, necessitating careful consideration.

In preclinical settings, specific behavioral impacts of fluoxetine have been more extensively studied. Its influence includes, reducing alcohol preference in rats (Naranjo et al., 1994), inhibiting muricidal behavior (Fuller, 1996), and decreasing the self-administration of substances like D-amphetamine (Yu et al., 1986) or cocaine (Glatz et al., 2002). Interestingly, fluoxetine has also been noted to enhance the analgesic properties of morphine (Barakat et al., 2018), showcasing its diverse array of effects in animal behavior**.** Specific behavioral effects of fluoxetine have been tested more frequently in preclinical models. This intricate network of fluoxetine’s interactions—spanning the spectrum from common side effects to specific behavioral changes—highlights the need for ongoing research to fully understand and effectively manage the drug’s diverse impacts.

## Concluding remarks and perspectives

The impact of fluoxetine on multiple cognitive domains are diverse, and there is evidence indicating potential advantages for memory and cognition across different conditions, although detrimental effects cannot be disregarded, especially in young subjects and in prenatal exposure to the drug. A second factor of disparity are variable baseline conditions in these studies, as fluoxetine may negatively affect the cognition of healthy brains (naïve animals), while improving diseased brains (e.g., including models of MDD, Alzheimer´s Disease, Traumatic Brain Injury, Obsessive-Compulsive Disorder, Stroke, Ischemia, Mild Cognitive Impairment, among others). For example, fluoxetine may affect positively especially depressive behavior and motor recovery after stroke, although its positive cognitive effects are still under discussion (Mead et al., 2020; Wu and Qin, 2023).

Special attention should be paid to evidence showing that perinatal fluoxetine administered to the mother may impair cognitive functions in the offspring, revealing a negative fetal programming action (Boulle et al., 2016a; Boulle et al., 2016b; Laureano-Melo et al., 2020; Lan et al., 2023).

**FIGURE 1:**
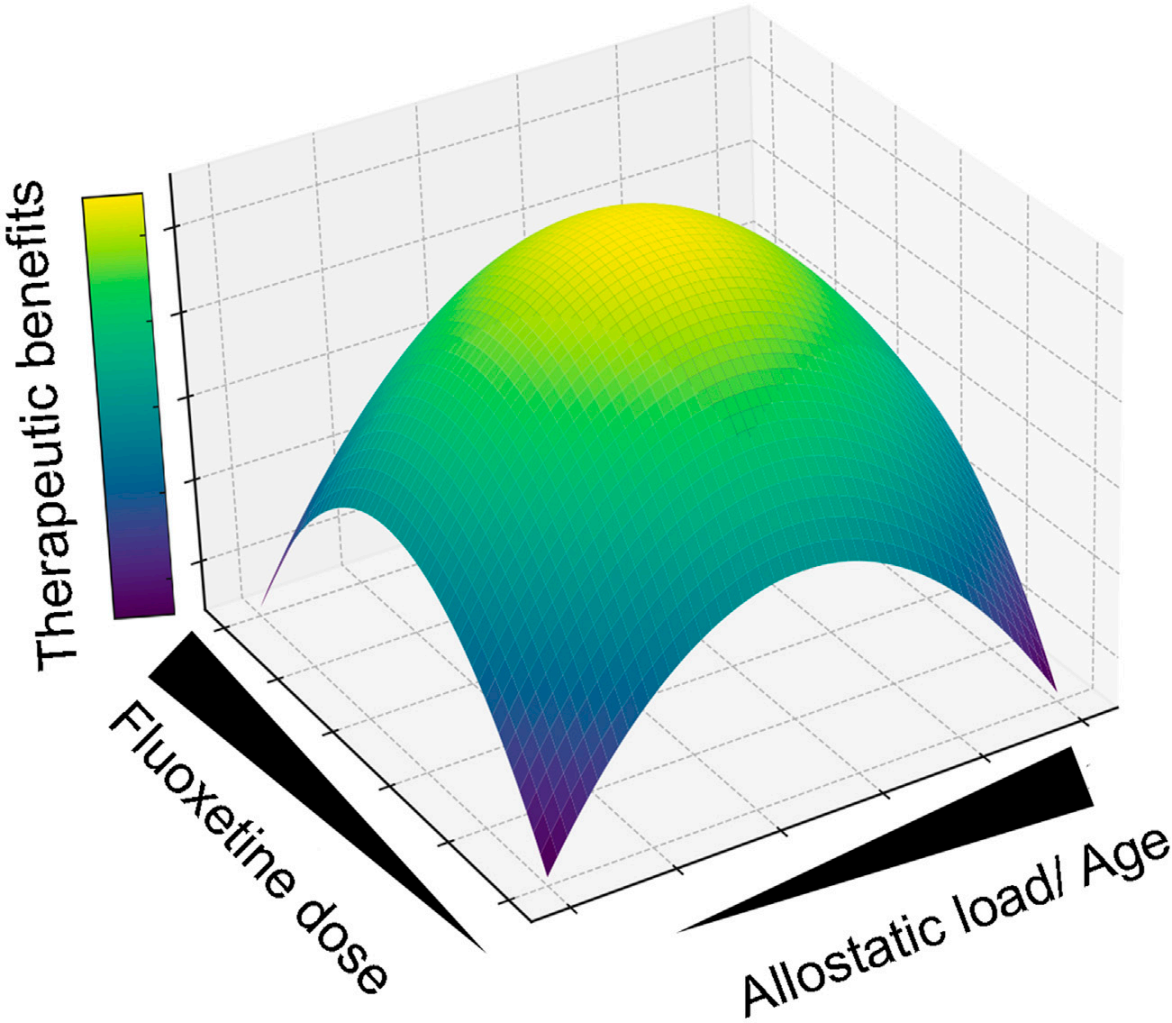
Multidimensional Model of Fluoxetine’s Therapeutic Response. This graphical abstract synthesizes the integrated preclinical and clinical findings, illustrating how the efficacy of fluoxetine therapy is modulated by allostatic load and dosing. The surface’s color gradient delineates the therapeutic optimum, pinpointing where allostatic load and fluoxetine dosage intersect to provide the greatest therapeutic advantage. For instance, comparable allostatic stress levels yield divergent therapeutic outcomes when fluoxetine is administered during different life stages; early life exposure (e.g., prenatal or adolescent phases) may be less beneficial than treatment in adulthood. Moreover, the therapeutic impact of a constant fluoxetine dose varies significantly with allostatic intensity, peaking at moderate levels (e.g., Mood disorders) while diminishing when levels are too low (Neurological/psychiatric homeostasis) or excessively high (Neurodegeneration).

It is difficult to predict the impact on human daily life of any of the reported alterations detected in preclinical studies, while complex cognitive skills are still very scarcely evaluated in any study. Considering these and other relevant preclinical and clinical findings from this article, we propose a multidimensional model of fluoxetine’s therapeutic response, which is dependent on the age, dose, and the level and nature of the allostatic load of the recipient organism (Figure 1).

Long-term consequences of fluoxetine have not adequately been addressed (e.g., after at least over 1 year of administration), especially when considering that it can be administered during long time periods. In a pioneering work including RNA-seq and H3K27ac ChIP-seq analysis of 27 brain regions after 6 weeks of fluoxetine treatment, the complexity and heterogeneity of fluoxetine-induced changes were addressed, opening a real possibility that the biological basis of antidepressant efficacy and adverse cognitive effects can be mechanistically explored (Rayan et al., 2022).

More recently, emerging evidence supports the anti-inflammatory role of fluoxetine. Indeed, central and peripheral inflammation may constitute a main etiological factor contributing to MDD (Beurel et al., 2020). In addition, neuroinflammation is consistently associated with cognitive impairment and with diseases that are risk factors for cognitive decline (Ownby, 2010). Interestingly, fluoxetine decreases neuroinflammation associated with various neurological disorders and inflammatory conditions, including MDD (Lee et al., 2012; Zhang et al., 2012; Du et al., 2016; Liu F. Y. et al., 2018). For instance, fluoxetine has anti-inflammatory properties in “healthy” rats subjected to a periodontal disease model (Branco-de-Almeida et al., 2012). Also, the cognitive impairment associated to neuroinflammation after surgery for knee removal of elderly patients can be effectively treated with fluoxetine (Lin et al., 2022). It remains unknown whether cognitive deficits are associated with differential patterns of neuroinflammation during MDD or even MDD subtypes (Lotrich, 2015; Gaspersz et al., 2017). On the other side, while anti-inflammatory drugs have been used successfully as add-on therapies for MDD (Zhang et al., 2016; Kohler-Forsberg et al., 2019), the associated eventually positive cognitive side effects have not yet been evaluated (Kohler et al., 2014).

To refine the therapeutic use of fluoxetine and even bolster our understanding of MDD pathogenic mechanisms, the following may contribute to guide future research:

How do the cognitive side effects of fluoxetine vary across different subtypes of depression, and what implications does this have for personalized treatment approaches?

What is the relationship between the age of patients and the manifestation of cognitive side effects, and how the age-specific treatment protocols would be developed?

How do underlying health conditions, both mental (such as MDD) and physical, interact with fluoxetine’s cognitive side effects, and what does this mean for comprehensive patient care?

Investigating these questions is crucial for tailoring fluoxetine use to individual needs, minimizing its risks, and harnessing its potential benefits. The insights gained from such research could revolutionize our approach to treating cognitive impairments associated with depression and inflammation.
