# Supplementary material for: The multifaceted effects of fluoxetine treatment on cognitive functions
Source: Front Pharmacol. 2024 Jul 16;15:1412420. doi: 10.3389/fphar.2024.1412420 (PMC11286485; doi:10.3389/fphar.2024.1412420)
Supplement: Supplementary file 1 [file Table1.pdf]

Preclinical Cognitive Domain	TEST	Description
Associative learning and memory	One-trial appetitive learning task	Evaluates the ability to learn and remember a task or behavior that leads to a rewarding outcome after a single exposure.
	Reward learning/Appetitive conditioning	Assesses the ability to learn associations between a stimulus and a rewarding outcome, often involving food or other rewards.
	AAT: Active avoidance Test	Measures the ability of rodents to learn and remember to avoid an aversive stimulus (e.g., a shock) by performing a specific action (e.g., moving to another compartment).
	IAT: Inhibitory Avoidance Test	Assesses the ability to learn and remember to avoid an area associated with an aversive stimulus, typically a foot shock
	CFC: Contextual/cued fear conditioning	Evaluates the association between a specific environment and an aversive stimulus, leading to a conditioned fear response when the animal is placed back in the same environment.
Spatial memory	OLM: Object Location Memory	Tests the ability to recognize and remember the locations of objects within an environment or arena
	MWM: Morris Water Maze	Assesses the ability to navigate and remember the location of a hidden platform in a pool of water using spatial cues.
	Barnes maze test	Measures the ability to learn and remember the location of an escape tunnel on a circular platform with multiple holes, using spatial cues.
Working memory	Y-maze	Assesses the ability to remember and navigate through a maze with Y- arms, testing memory and exploratory behavior.
Recognition memory	NOR: Novel Object Recognition	Measures the ability to recognize a novel object in contrast to a familiar one, testing memory and recognition processes.
Impulse control and decision-making	Impulsivity test (Wisconsin)	Measures the tendency to make impulsive choices, often involving delay discounting or preference for immediate rewards over delayed rewards.
Attention, arousal, and cognitive processing speed	Pupil size and reaction time in visual test	Assesses changes in pupil size and reaction times in response to stimuli, providing insights into attentional and cognitive processing mechanisms in a visuo-spatial test.

Attention and vigilance	CPT: Continuous Performance Test	Tests the ability to sustain attention and respond to specific stimuli over a period, often involving the detection of target stimuli among non-targets.
Anxiety and avoidance learning	Open arm escape	Evaluates the tendency to escape from an aversive or anxiogenic environment, often using a maze with open and closed arms.
Neurological assessment	Stroke-index McGraw scale	Provides a quantitative measure of motor and neurological deficits following a stroke, assessing the extent of impairment and recovery.
Cognitive flexibility and spatial learning	Place learning reversal	Tests the ability to adapt to changes in the environment, such as learning a new location for a reward after the original location has been changed.
Reward processing and addiction	Cocaine or amphetamine self administration	Measures the tendency to self-administer drug, assessing the reinforcing effects of the drug and the neurobiological basis of addiction.

Clinical Cognitive Domain	Test	Description	Similar to preclinical tests
Memory function	PGIMS: The PGI Memory Scale	Measures different aspects of memory function, including immediate and delayed memory, recall, recognition, and retention.	*
Memory function	WMS-III: Wechsler Memory Scale III	Evaluates different aspects of memory, including immediate and delayed recall, auditory and visual memory, and working memory	*
Executive function and prospective memory	SLT: Shopping List Task	Assesses the ability to remember and execute tasks related to a list, simulating real-world memory demands.	
Self-reported memory function	SMQ: Subjective Memory Questionnaire	Evaluates an individual's perception of their memory capabilities and difficulties in daily life.	
Executive function and visuospatial ability	TPC: Ten-Point Clock Drawing score	Assesses the ability to understand and reproduce a clock face, testing spatial organization and planning.	

Episodic memory	USC-REMT: University of Southern California Repeatable Episodic Memory Test	Measures the ability to recall and recognize information from specific past events.	
Executive function and language	VFT: Verbal Fluency Test	Assesses the ability to generate words within a specific category (semantic fluency) or starting with a specific letter (phonemic fluency) within a limited time.	
General intelligence	WAIS-III: Wechsler Adult Intelligence Scale	Comprehensive test measuring various cognitive abilities, including vocabulary similarities and arithmetic capabilities	
Various memory functions	WMS-III: Wechsler Memory Scale III	Comprehensive test measuring various cognitive abilities, including verbal comprehension, perceptual reasoning, working memory and processing speed.	*
Non-verbal cognitive ability	WNV: The Wechsler Non-verbal Scale of Ability	Measures cognitive ability without relying on language, assessing skills such as problem-solving and visual-motor coordination.	
Associative memory	WPW: Wechsler Paired Word Test	Tests the ability to remember and recall pairs of words, measuring associative learning and memory.	*
General cognitive function	MMSE: Mini-Mental State Examination	Brief screening tool for assessing cognitive impairment, covering areas such as orientation, attention, memory, language, and visuospatial skills.	*
Cognitive and behavioral assessment	CLAS: Clifton Assessment Schedule	Measures cognitive function and behavioral symptoms, often used in elderly populations to assess dementia-related changes.	*
Attention and visual scanning	CTT: Cancellation Task Test	Measures the ability to maintain attention and accurately scan and mark specific items in a visual array.	*
Sustained attention and vigilance	CPT: Continuous Performance Test	Assesses the ability to maintain consistent attention and respond to target stimuli over a prolonged period.	*
Processing speed and working memory	DSST: Digit Symbol Substitution Test, WAIS subtest	Measures the speed of processing and the ability to use a key to substitute symbols for digits, assessing cognitive	*
Memory and spatial orientation	BIMT: Blessed Information and Memory Test	Measures memory and orientation, particularly in elderly populations.	*
Verbal memory	BSRT: Buschke Selective Reminding Test	Assesses the ability to remember and recall words through repeated learning trials, measuring long-term storage and retrieval.	

Various cognitive domains.	CANTAB: Cambridge Neuropsychological Test Automated Battery	A computerized test battery that assesses multiple cognitive functions, including memory, attention, executive function, and visuospatial skills.	*
Decision-making and impulse control	Experimental drinking sessions	These sessions are used to study the cognitive and behavioral effects of alcohol consumption, examining aspects such as decision-making, risk-taking, and impulse control under the influence of alcohol. Participants' drinking behavior, including patterns of consumption and responses to cues, can provide insights into cognitive processes and potential vulnerabilities related to alcohol use.	*
Executive function, processing speed, and cognitive flexibility.	Trail Making Test	The test is divided into two parts: Part A: assesses visual attention and processing speed by requiring individuals to connect numbered circles sequentially. Part B: assesses cognitive flexibility and task switching by requiring individuals to alternate between numbers and letters in sequence (e.g., 1-A-2-B-3-C). The time taken to complete each part and the number of errors is recorded, providing information on executive function and the ability to shift attention.	*
Sustained attention and vigilance.	Mackworth Clock Test	This test assesses an individual's ability to maintain attention and detect infrequent signals over a prolonged period. Participants are asked to monitor the movement of a clock hand and respond to irregular jumps or missed beats. The test measures the decline in vigilance over time and is often used in research on attention, fatigue, and the effects of various factors (e.g., sleep deprivation, medications) on sustained attention.	*
